# Supplementary material for: A UV/Vis Spectroscopy-Based Assay for Monitoring of Transformations Between Nucleosides and Nucleobases
Source: Methods Protoc. 2019 Jul 15;2(3):60. doi: 10.3390/mps2030060 (PMC6789650; doi:10.3390/mps2030060)
Supplement: Supplementary file 1 [file mps-02-00060-s001.zip › Supplementary_Information__Kaspar_Giessmann_et_al_2019.pdf]

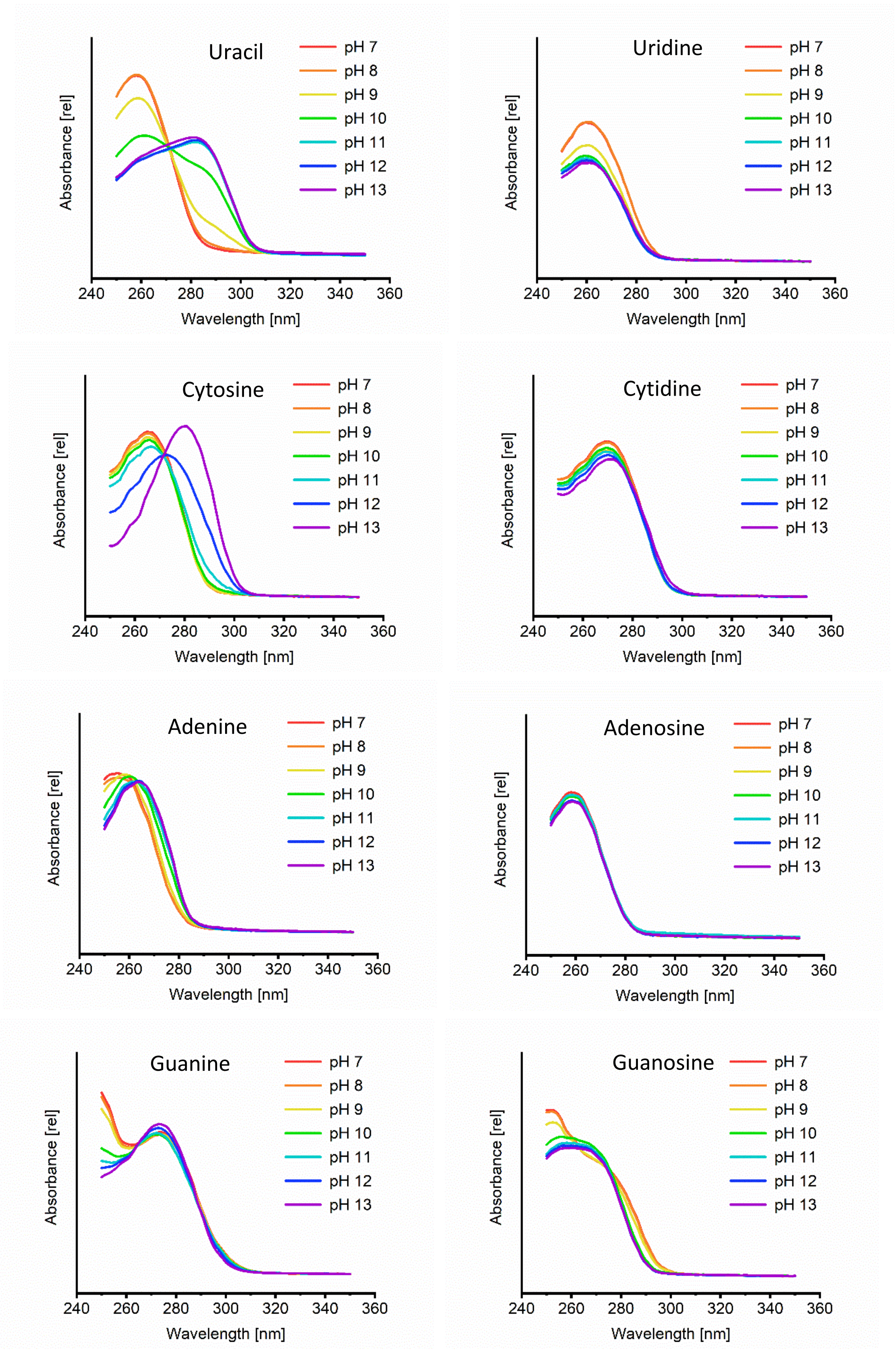
Uracil
Uridine
Cytosine
Cytidine
Adenine
Adenosine
Guanine
Guanosine
pH 7
pH 8
pH 9
pH 10
pH 11
pH 12
pH 13
Absorbance [rel]
Wavelength [nm]
240
260
280
300
320
340
360

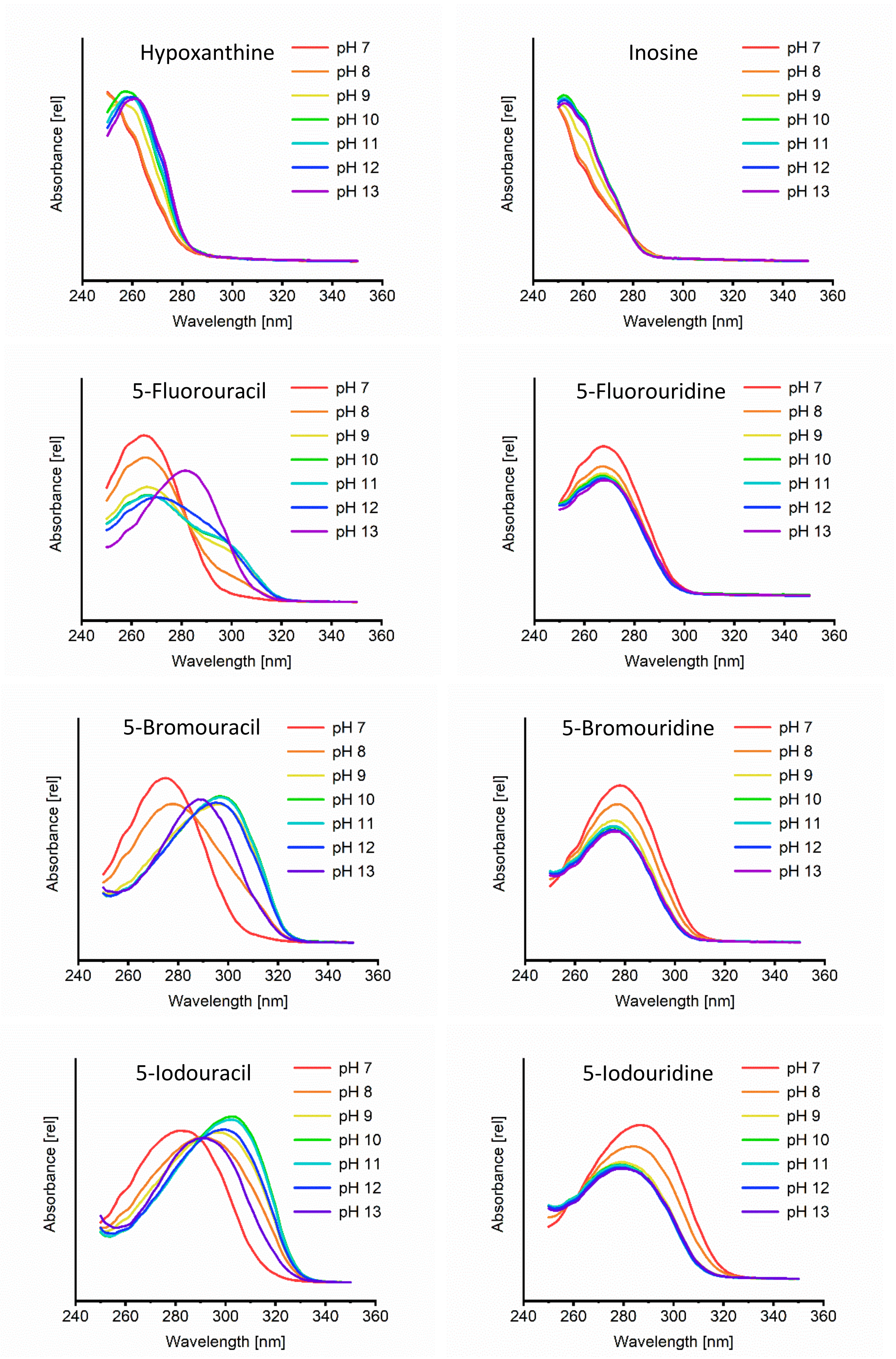
Hypoxanthine
Inosine
5-Fluorouracil
5-Fluorouridine
5-Bromouracil
5-Bromouridine
5-Iodouracil
5-Iodouridine
pH 7
pH 8
pH 9
pH 10
pH 11
pH 12
pH 13
Absorbance [rel]
Wavelength [nm]
240
260
280
300
320
340
360

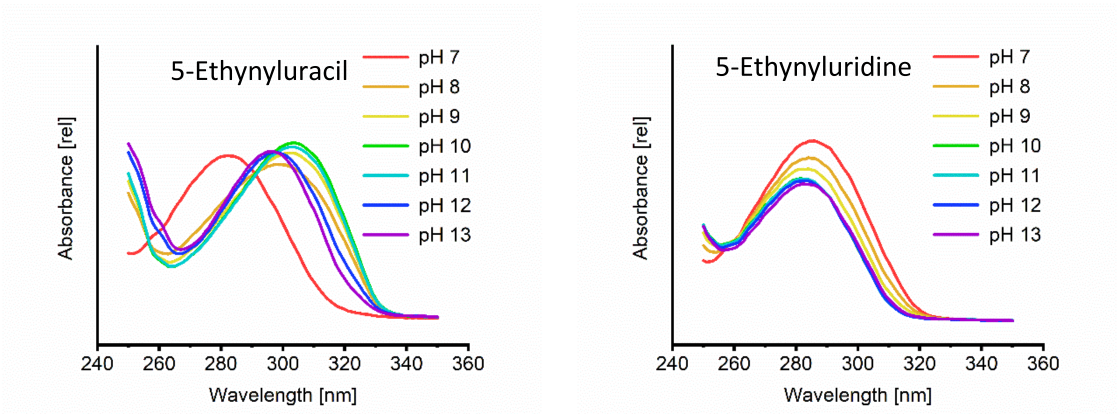

Figure S1 pH dependence of the UV/Vis-absorption spectra of the other nucleosides in this study.

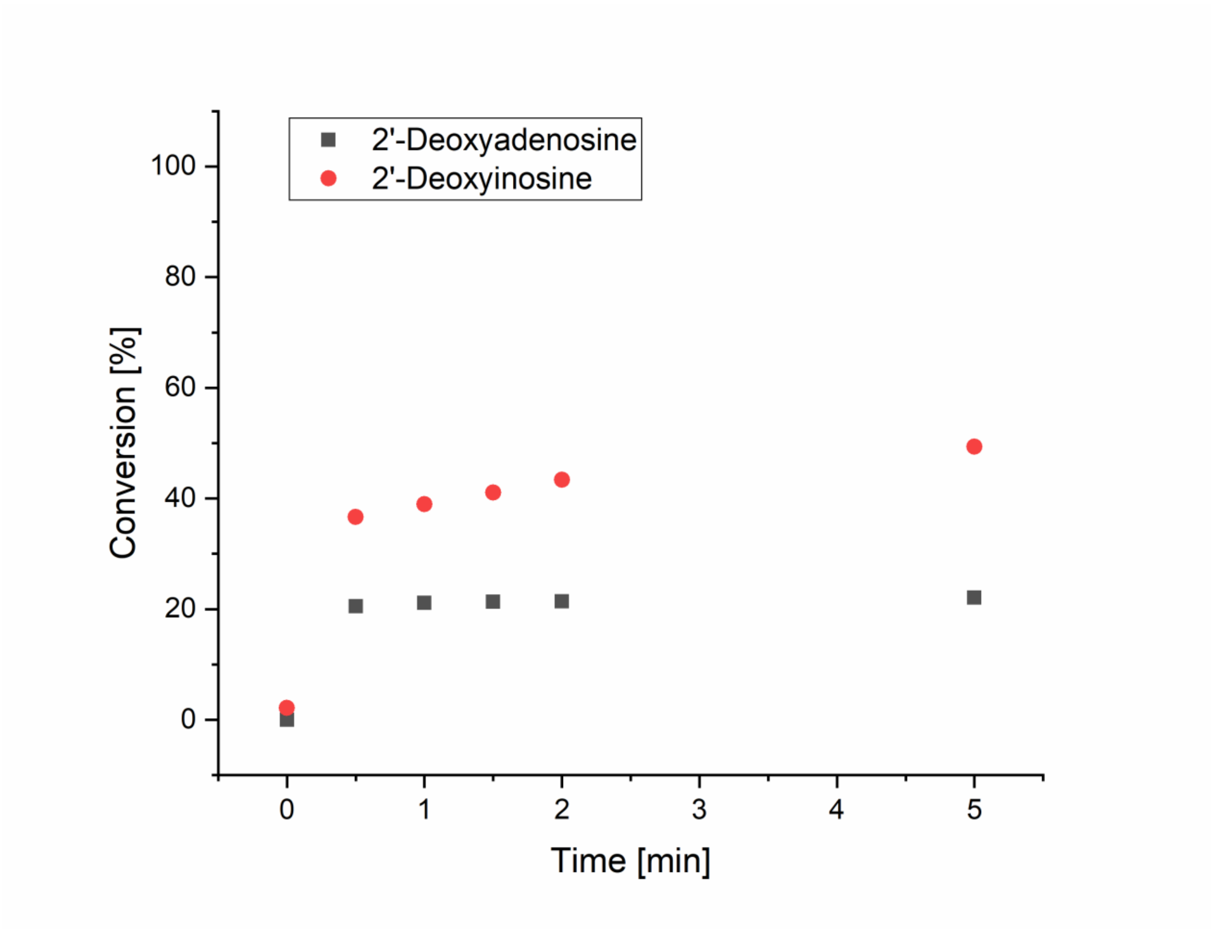

Figure S2 Phosphorylysis of 2'-deoxyadenosine and 2'-deoxyinosine by Pu-NPase. The reactions were performed with 2 mM nucleoside, 10 mM K_2HPO_4, 16 µg/mL Pu-NPase in 50 mM glycine buffer at pH 9 and 60 °C.
